# Spatial spillover and threshold effect of green development efficiency on urban human settlement resilience in Yangtze River Delta urban agglomeration

**DOI:** 10.1371/journal.pone.0292230

**Published:** 2023-10-19

**Authors:** Zheng Wang, Xiaobo Xu, Jie Zhang

**Affiliations:** School of Public Policy and Management, Anhui Jianzhu University, Hefei, Anhui, China; Shanghai Dianji University, CHINA

## Abstract

Green development is the necessary path and fundamental way for urban development. Exploring the influence mechanism and spatial effect of green development on the urban human settlement resilience is conducive to promoting high-quality and sustainable urban development. We used the entropy method, super-efficient SBM model, spatial econometric model and threshold model to analyze the spatial spillover effect of green development efficiency on urban human settlement resilience and its nonlinear impact in the Yangtze River Delta(YRD) urban agglomeration. The results indicated that During the study period, the level of green development efficiency and urban settlement resilience was on the rise, and the spatial differences between different regions was significant. The green development efficiency of each city in the YRD urban agglomeration has a significant contribution to urban human settlement resilience in the region, but has a negative spatial effect on the level of urban human settlement resilience in the neighboring region. At different population density levels, the effect of green development efficiency on urban human settlement resilience shows a significant "V" non-linear characteristic. Furthermore, the influence of green development efficiency on urban human settlement resilience increases in a stepwise manner under different industrial structure distribution. The results of this study can help provide a reference basis for the creation of high-level, high-quality green and livable resilient cities in the YRD urban agglomeration under the concept of green development, and provide relevant experience for the construction of livable cities in other regions of China.

## 1. Introduction

Cities have become increasingly critical in today’s society, serving as the central engine of social development and providing residents with broader development opportunities and more diverse life experiences in the accelerating process of economic globalization and urbanization [1]. However, the rapid growth of cities has brought about a rapid expansion of regional population scale and geographic space [2]. These changes have led cities to face challenges such as environmental pollution [3,4], disease epidemics [5], and resource depletion [6], which have profoundly altered the human habitat as an important living space. As a result, research on green development approaches to enhance urban human settlement resilience has gradually emerged, which aims to explore effective ways for cities to cope with complex environmental challenges, ensuring the quality of life for residents and social stability, and ultimately promoting the urban development to be more sustainable and resilient.

Urban human settlement resilience refers to the ability of human settlement systems to withstand natural disasters or social crises, as well as to recover from such challenges, which reflects the level of urban human settlement construction and its potential for sustainable development. From the development history of the concept, the discussion of urban human settlement resilience originated within the field of human settlement studies in the late 19th century, and its focus and conceptual connotations subsequently changed at different times of urban development. As the connotation of urban human settlement and related theories have been clarified, the direction of urban development under the people-oriented perspective has begun to become a consensus, which holds that urban construction should focus not only on the planning and design of urban buildings, but also on serving human development. Based on this, Howard E. proposed garden cities, in which he argued that there should be a reasonable proportion of residential, industrial, and agricultural areas, and that human communities should be surrounded by areas of fields or gardens [7]. Register R, after studying traditional villages and rural areas, argued that cities should be reconstructed in pursuit of healthy human and natural vitality, and categorized this theory as ecological cities [8]. Wu proposed the concept of human settlement science by focusing on the demands and aspirations of community residents during urban development [9], which represents the rise of human settlement science in China.

Overall, these studies are actually centered on the relationship between green development and urban construction, which enables modern cities to provide basic living conditions and suitable living environments through innovative planning of new development models for cities. It is worth noting that, as the process of urbanization enters a new phase, the human living environment is gradually changing, which means that urban development is already facing the impact and superimposition of multiple risks, with the frequent occurrence of natural disasters seriously affecting the safety of residents’ lives and the quality of urban development. In this context, the concept of "resilience", referring to the capacity to restore a normal state following a disturbance, has been incorporated into urban planning research. It is evident that the growing contrast between ecological environment degradation and the demands of urban development is increasingly conspicuous [10].

As a result, "green development" has become an important direction for future urban development. At the policy level, during the Fifth Plenary Session of the 18th CPC Central Committee, the five development concepts of "innovation-driven, coordinated, green, oriented toward global progress, and beneficial to all" were proposed for the first time, providing guidance for the green development model of cities. In the practice of urban renewal and development, urban green transformation research has received more attention, so that the 20th Party Congress report further emphasizes the need to “accelerate the green transformation of development methods, to create a livable, resilient, and intelligent city”.

The existing research on the impact of green development on urban construction has mainly focused on two aspects. One aspect involves studying the connection between the urban economy and ecological resources from the perspective of urban ecology planning [11,12]. For example, Zeng et al. directed their attention to the process of green development to assess the current status of green development in urban areas [13]. Yan analyzed the upgrade path of the city’s industrial structure based on the perspective of green technology innovation [14]. In contrast, The second aspect centers on investigating the impact of green development on social welfare and the urban living experience, which argues that ecological livability is a vivid manifestation of social development [15,16], opposes the "short-sighted" approach of quick fixes, and emphasizes the importance of infrastructure development [17], ecological preservation and upgraded consumption patterns to ensure that urban residents are able to enjoy the benefits of green development [18].

In conclusion, green development and urban human settlement resilience have been studied from various perspectives, providing a valuable preliminary reference for this study. However, there are several practical matters that warrant more thorough exploration. For instance, how does green development contribute to urban human settlement resilience? Does this impact exhibit spatial heterogeneity or a threshold interval effect? Addressing these issues could lead to innovations and breakthroughs in the study of urban human settlement resilience, enhancing our understanding and perception of the concept.

To address these questions, mathematical and statistical methods will be employed to examine the spatial-temporal evolution of urban human settlement resilience in the YRD urban agglomeration. Specifically, we will construct spatial panel regression models and threshold regression models to uncover the time-series characteristics. Additionally, the focus will be on investigating the spatial and threshold effects of green development on urban human settlement resilience. This study aims to enrich the theoretical knowledge of urban human settlement resilience and green development, providing valuable and sustainable policy references for urban planning.

## 2. Mechanism analysis

**Fig 1:**
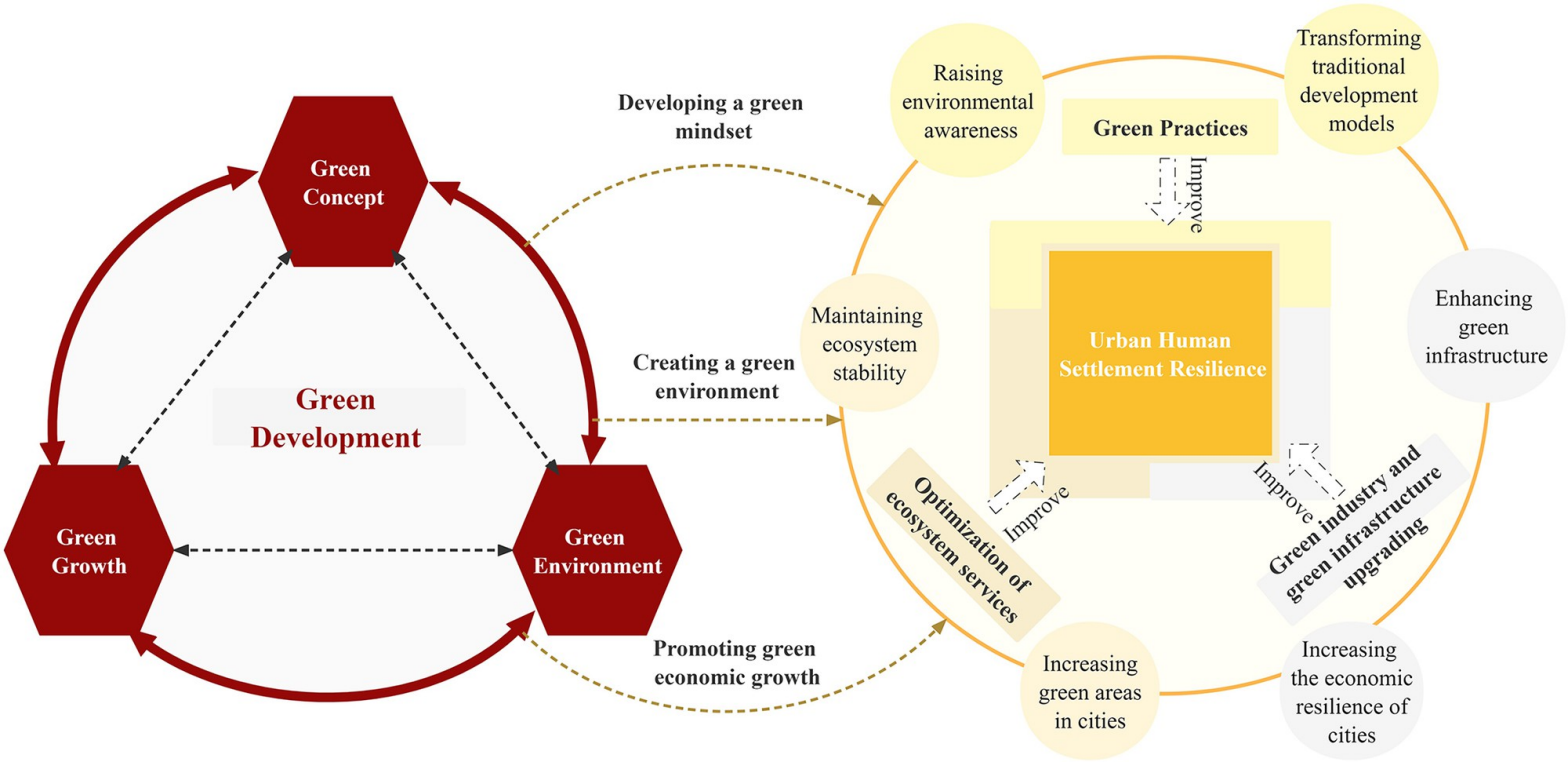
Mechanism map for green development impacts on urban human settlement resilience.

The core principle of green development was the envisioned harmonious coexistence between human beings and nature, which seeks to foster a positive cycle of economic growth and environmental preservation, leading to mutually beneficial outcomes that encompass both economic and environmental advantages [19]. According to the mechanism of action, green development has both "pollution abatement" and "economic growth" effects. This means that green development can promote urban human settlement resilience through a complex system of mutual applications, feedbacks and links between human activities and the natural environment. Therefore, we clarified the mechanism of green development on urban human settlement resilience from the dimensions of green concept, green environment and green growth, and produced th mechanism analysis graph to provide a more intuitive representation (Fig 1).

1. Mechanisms of action of the green concept on enhancing urban human settlement resilience. Symbiosis theory suggests that a mutually beneficial relationship of production and exchange will be formed between urban development and the ecological environment, so urban development should be realized with the premise of protecting the ecological environment [20]. Based on this, the widespread acceptance of the green concept has the potential to change the traditional development thinking associated with uncontrolled urban expansion, which could help to raise the environmental behavior of residents, thereby facilitating the social construction of a green ecological civilization and ultimately the promotion of urban human settlement resilience [21].
2. Mechanisms of action of the green environment on enhancing urban human settlement resilience. The theory of ecosystem services emphasizes the important value of natural ecosystems to human society, which argues that ecosystem services can be categorized into supportive, regulating, provisioning, and cultural services [22]. Specifically, green development can not only maintain the supportive and regulating services of ecosystems by stabilizing them and establishing natural protection systems, but also strengthen the provisioning and cultural services of ecosystems by increasing green space in cities. As cities establish stable and diverse ecosystem services, their dependence on external resources will gradually decrease, and thus urban human settlement resilience can be enhanced.
3. Mechanisms of action of the green growth on enhancing urban human settlement resilience. The environmental Kuznets curve demonstrates that increasing levels of green economic development promote the upgrading of green industries in cities, creating more jobs and improving the economic efficiency of cities. As a result, green economic growth can provide cities with a more stable basis for economic growth, increasing their economic diversity and reducing their dependence on a single industry [23]. In addition, actively developing green industries and promoting clean energy during urban development can reduce the impact of environmental pollution and external shocks on the economic stability of cities [24].

## 3. Methods and data

### 3.1 Selection of study area

**Fig 2:**
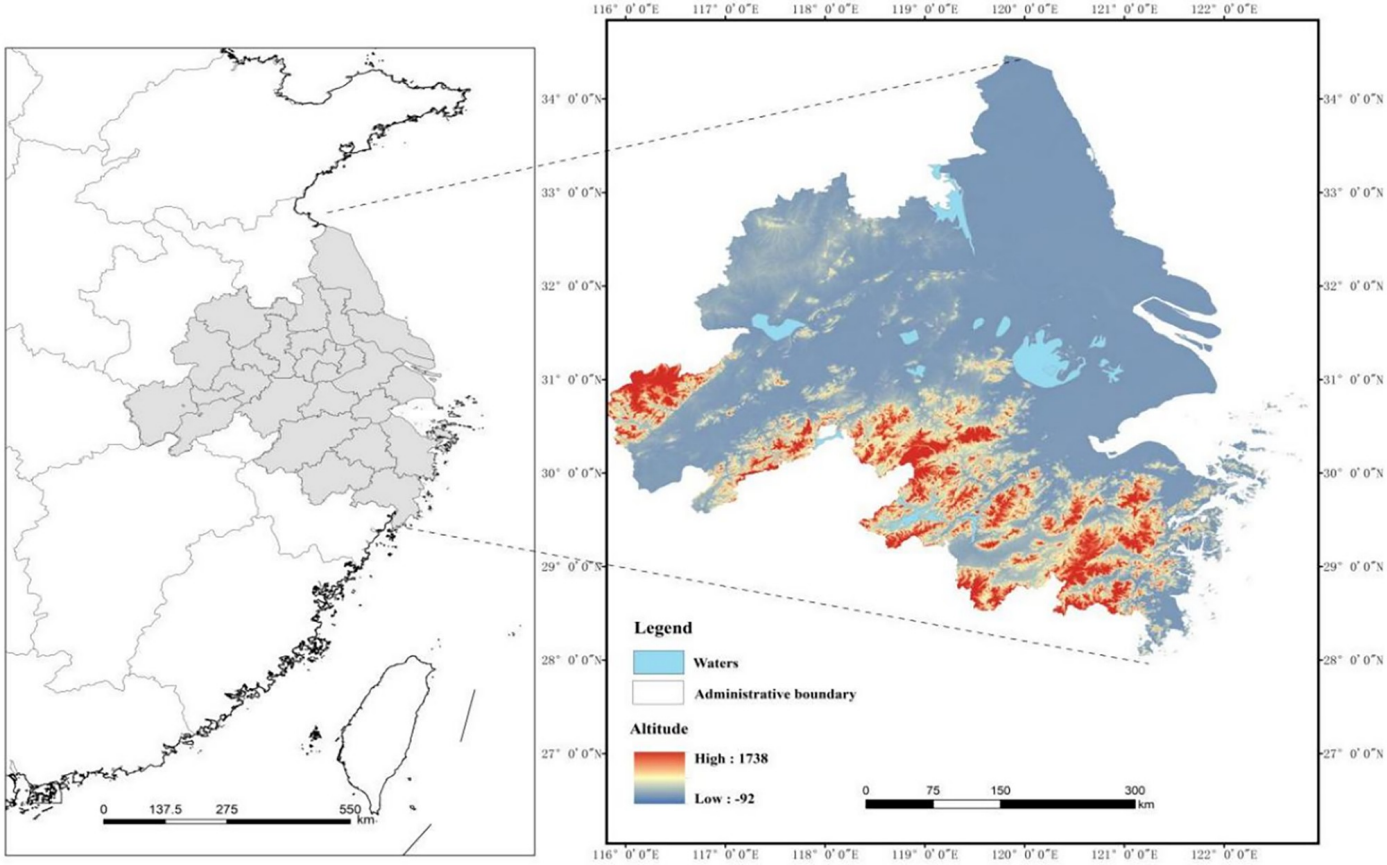
The study area. **Note**: The base map of the spatial analysis map used in the study was obtained from the website of the Standard Map Service of the Ministry of Natural Resources, with the review number GS (2019) 3333, and the base map was not modified.

The YRD urban agglomeration is important as one of the rapidly developing industrialized regions in China, as well as a key region and strategic supporting point for China’s economic growth. Furthermore, it is a demonstration area for implementing the development strategy of "ecological priority and green development". Geographically, the YRD urban agglomeration is situated in the alluvial plain formed by the estuary of the Yangtze River, which is the largest and most dynamic economic zone in China and has an important strategic position in the eastern coastal region (Fig 2). More importantly, the YRD urban agglomeration exhibit notable variations in development conditions, economic foundations, and resource allocation, consequently giving rise to regional disparities in their development levels and constraints. These divergent development patterns render the practical experiences and outcomes of the YRD urban agglomeration valuable not only as a guide for other developed regions, but also as a blueprint for backward regions.

### 3.2 Data sources

**Fig 3:**
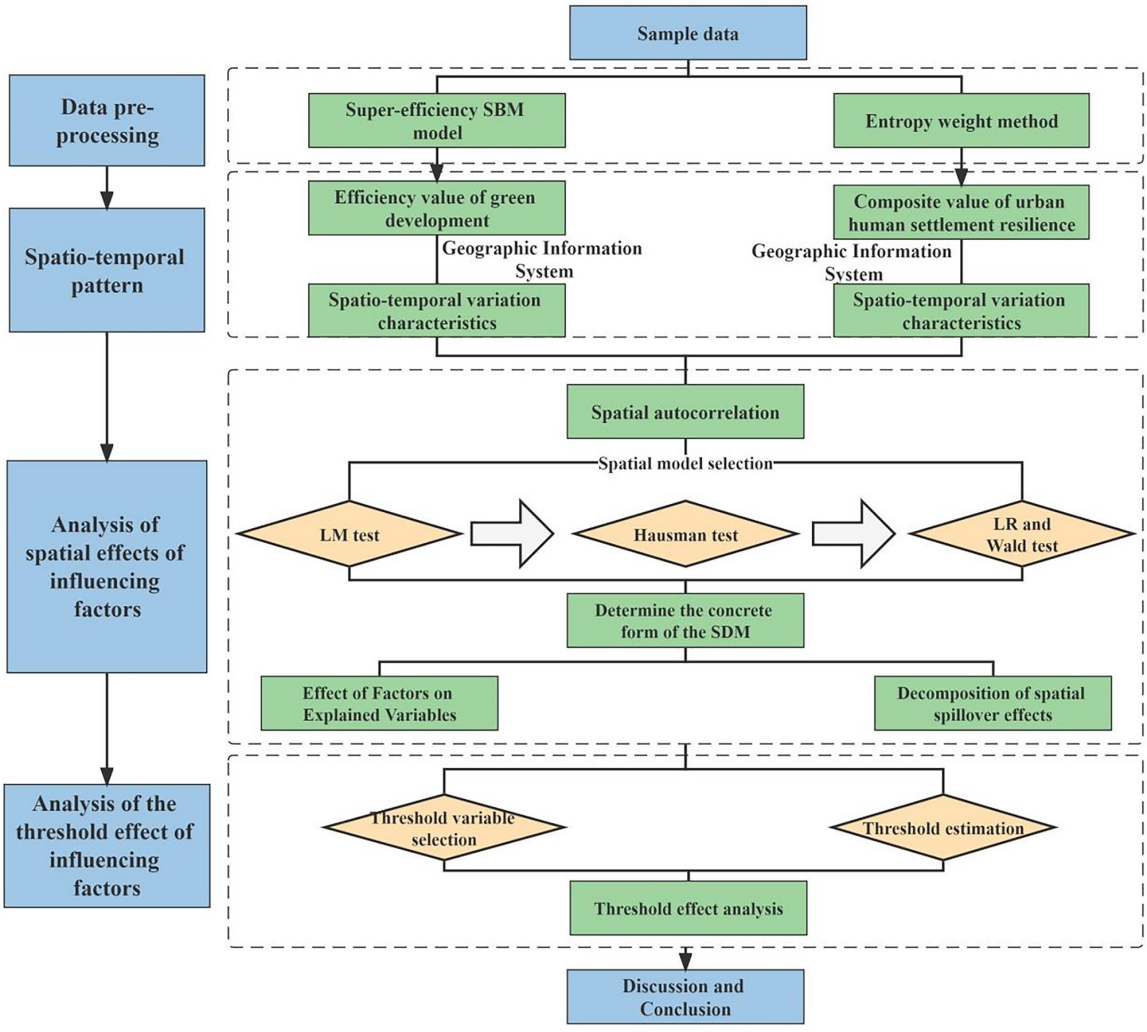
Design of research methods.

Considering the data updates, the relevant data of 26 cities in the YRD urban agglomeration from 2010–2020 are used to measure the spatial and nonlinear effects of green development efficiency on urban human settlement resilience in the region. The data are mainly from the Chinese Statistical Yearbook, the Shanghai Statistical Yearbook, the Jiangsu Statistical Yearbook, the Anhui Statistical Yearbook, the Zhejiang Statistical Yearbook, the statistical yearbooks of various cities, as well as the statistical bulletin on national economic and social development. The main workflow is shown in Fig 3.

### 3.3 Research methods

#### 3.3.1 Entropy weight method

The entropy weighting method is a traditional objective weighting method that calculates the weight coefficients of indicator weights based on the dispersion of each indicator, which can avoid the subjectivity of artificial assignment [25]. This study adopts the entropy weight method to measure the level of urban human settlement resilience in cities within the YRD urban agglomeration. The specific steps are as follows:

The first step is data normalization:

Positive indicator:

$$x_{ij}^{\prime }=\frac{x_{ij}-\;min\;x_{j}}{max\;x_{j}\;-\;min\;x_{j}}$$

Negative indicator:

$$x_{ij}^{\prime }=\frac{max\;x_{j}\;-\;x_{ij}}{max\;x_{j}\;-\;min\;x_{j}}$$

Neutral indicators:

$$x_{ij}^{\prime }=\frac{max\left\{\left|x_{ij}-\;x_{j}^{*}\right|\right\}\;-\left|x_{ij}-x_{j}^{*}\right|}{max\left\{\left|x_{ij}-x_{j}^{*}\right|\right\}\;-\;min\left\{\left|x_{ij}-x_{j}^{*}\right|\right\}}$$

Where $x_{ij}^{\prime }$ refers to the value of the *j* th indicator in unit *i* and $x_{ij}^{\prime }$ is still denoted as *x*_*ij*_ in this study; $x_{j}^{*}$ is the mean value of the *j* th indicator.

The second step is to calculate the entropy value:

$$e_{ij}=\;-k\sum_{i=1}^{m}(\frac{x_{ij}^{\prime }}{\sum_{i=1}^{m}x_{ij}^{\prime }}\;\cdot \;ln\;\frac{x_{ij}^{\prime }}{\sum_{i=1}^{m}x_{ij}^{\prime }})k\;=\frac{1}{ln\;m}$$

The third step is to calculate the weight:

$$w_{j}=\;\frac{1-e_{j}}{\sum_{j=1}^{n}\left(1-e_{j}\right)}$$

The last step is to calculate the comprehensive score:

$$s_{j}=\sum_{j=1}^{n}w_{j}\cdot x_{ij}^{\prime }$$

#### 3.3.2 Super-efficiency SBM model

The Data Envelopment Analysis (DEA) method was jointly proposed by Charnes, Cooper, and Rodes in 1978, providing a nonparametric approach to estimating the efficiency of multiple decision units. Building upon the traditional DEA model, Tone introduced the super-efficient SBM model to address the slack variable issue and enable the evaluation of multiple decision efficient units. Therefore, the super-efficient SBM is employed in this study to measure the green development efficiency of the YRD urban agglomeration [26]. The formulas are as follows:

$$\begin{array}{c} \text{min}\;\delta =\frac{\text{1}+(\frac{1}{m})\sum_{i=1}^{m}\frac{w_{i}^{-}}{x_{ik}}}{\text{1-(}\frac{1}{s})\sum_{r=1}^{s}\frac{w_{r}^{+}}{y_{rk}}}\\ \begin{array}{cc} s.t & x_{ik}\geq \sum_{j=1,j\neq k}^{n}\lambda_{j} \end{array}x_{ij}-w_{i}^{-};y_{rk}\leq \sum_{j=1,j\neq k}^{n}\lambda_{j}y_{rj}+w_{r}^{+};\\ {\lambda_{j}\geq 0;w}_{i}^{-}\geq 0,i=1,2\ldots ,m;w_{r}^{+}\geq 0,j=1,2\ldots ,n; \end{array}$$

Where $w_{i}^{-}$ and $w_{i}^{+}$ refer to the input and output slack variables; *λ*_*j*_ is the constraint; *x*_*ik*_ and *y*_*rk*_ denot input indicators and output indicators, respectively; *δ* is the green development efficiency value, with higher *δ* representing higher efficiency.

#### 3.3.3 Spatial autocorrelation

Spatial autocorrelation analysis can be used to identify the spatial interdependence among elements within a geographic region. The Global Moran’s I is a common method for spatial autocorrelation analysis, which is used to reveal the overall spatial correlation within the study area [27]. Generally, it takes values in the range of [-1,1], while If it is greater than 0, it is positively correlated in spatial distribution; and if it is less than 0, it is negatively correlated. This study used Global Moran’s I to reveal the spatial agglomeration characteristics of green development efficiency and urban human settlement resilience in the study area, which is calculated as follows:

$$\text{Moran}\prime \text{s}\;I=\frac{\sum_{i=1}^{n}\sum_{j=1}^{n}w_{ij}\left(X_{i}-\bar{x}\right)\left(X_{j}-\bar{x}\right)}{\sigma^{2}\sum_{i=1}^{n}\sum_{j=1}^{n}w_{ij}}$$

Where $\sigma^{2}=\frac{1}{n}\sum_{i=1}^{n}{\left(X_{i}-\bar{x}\right)}^{2};\bar{x}=\frac{1}{n}\sum_{i=1}^{n}X_{i}$; *w*_*ij*_ is the spatial weight matrix; *X*_*i*_ and *X*_*j*_ denote the observationsin study unit *i* and study unit *j*.

#### 3.3.4 The spatial Durbin model

Considering that the YRD urban agglomeration has become an interconnected system, which the development of each region might have a cross-regional impacts on neighboring areas. Therefore, it is essential to examine spatial patterns and identify the factors influencing these patterns [28]. In this study, the spatial Durbin model (SDM) was utilized for this purpose. The model is as follows:

$$Hsr_{it}=a_{0}+\rho \sum w_{ij}Her_{it}+LnGde_{it}a_{1}+\Phi \sum w_{ij}LnGde_{it}+\eta X_{it}+\beta \sum w_{ij}X_{it}+\mu_{i}+\theta_{t}+\varepsilon_{it}$$

Where *Hsr*_it_ is the explained variable; *a*_0_ refers to the constant term; *X*_*it*_ represents the control variable; *ρ*, *Φ* and *β* are the spatial lag coefficients of the corresponding variables, respectively; *a*_1_ is the core explanatory variable regression coefficient; *μ*_*i*_ and *θ*_*t*_ represent spatial and temporal fixed effects, respectively; *ε*_*it*_ represents the random interference term; *w*_*ij*_ is the spatial weight matrix, and nested matrices were constructed for each interval with reference to Hao et al. and Xiao-Sheng L et al., setting *φ* = 0.5 [29,30] (Table 1).

**Table 1:** Weight matrix description.

Spatial weight matrix	Meaning	Calculation formula	Description
**Geospatial weight matrix *w*_1_**	Geographical distance between cities	$w_{1}=\frac{1}{d_{ij}}$	*d*_ij_ represents the longitude and latitude distance between city i and city j
**Economic weight matrix *w*_2_**	Economic disparities between provinces	$w_{2}=\frac{1}{\left|P_{i}-P_{j}\right|}$	P_*i*_ and P_*j*_ denote the mean values of real GDP per capita in province i and province j during the sample period
**Nested weight matrix *w*_3_**	Geographical proximity and economic disparity between provinces	*w*_3_ = *φ · w*_1_ + (1 − *φ*) *w*_2_	Product of adjacency matrix and economic matrix

#### 3.3.5 Panel threshold model

The "threshold effect" is based on the threshold estimates to examine the effect of each variable on the explained variables in different ranges, which takes into account the dynamic change process among variables more than simple linear regression [31]. In light of this, this study used the threshold model to explore the effect of green development efficiency on urban human settlement resilience at different levels of threshold variables, and then the formulas are as follows:

$$\begin{array}{ll} Hsr_{it} & =a_{0}+a_{1}\;Lngde_{it}I\left(pd\leq \gamma \right)+a_{2}\;Lngde_{it}I(pd>\gamma )+\beta X_{it}+\varepsilon_{it}\\ Hsr_{it} & =a_{0}+a_{1}\;Lngde_{it}I\left(is\leq \gamma \right)+a_{2}\;Lngde_{it}I(is\;>\;\gamma )+\beta X_{it}+\varepsilon_{it} \end{array}$$

Where *PD* and *IS* refers to the threshold variable; *γ* is unknown threshold values to be estimated; *I (·)* is an indicator function, and the rest of the variables have the same meaning as above.

### 3.4 Construction of indicator system and selection of variables

#### 3.4.1 Explained variable

The Pressure-State-Response (PSR) framework, originally proposed by Canadian statistician Friends in the late 1979s and further developed by the Organization for Economic Cooperation and Development (OECD) and the United Nations Environment Programme (UNEP) in the late 1980s, is widely employed to study urban problems [32,33]. According to the connotation of urban human settlement resilience [34,35], an evaluation index system (Table 2) was constructed to measure urban human settlement resilience based on the principles of systematicity, scientificity and comparability, which was divided into three dimensions: pressure (P), state (S) and response (R).

**Table 2:** Evaluation index system of urban human settlement resilience.

Layer of Target	Criterion Level	Index Level	Standard of measurement	Weights	Attribute
Urban human settlement resilience	pressure (P)	Natural Environment	Average annual temperature/°C	0.013	#
			Annual average relative humidity/%	0.015	#
			Average annual light hours/h	0.014	#
		Living Environment	Average regional ambient noise level/db	0.018	-
			Parkland area per capita/m^2^	0.019	+
		Urban Safety	Number of traffic fatalities per 10,000 people/people	0.001	-
			Incidence of fire in population/%	0.012	-
			Urban unemployment rate/%	0.008	-
	state (S)	Urban Infrastructure	Number of buses owned per 10,000 people/vehicle	0.082	+
			Road freight volume/t	0.067	+
			Library collections per capita/volume	0.095	+
			Density of drainage pipes in built-up areas/km	0.020	+
			Urban road area per capita/m^2^	0.021	+
		Technology Education	Number of patents granted/piece	0.116	+
			Number of college students per 10,000 people/people	0.141	+
		Health Care	Number of doctors per 10,000 people/people	0.027	+
			Number of medical beds per 10,000 people/piece	0.022	+
	response (R)	Society input	Environmental protection expenditure as a proportion of fiscal expenditure/%	0.050	+
			Education spending as a share of fiscal expenditure/%	0.017	+
			Science and technology spending as a proportion of fiscal expenditure/%	0.047	+
		Environmental Governance	Centralized wastewater treatment rate/%	0.015	+
			General industrial solid waste utilization rate/%	0.008	+
			Garbage disposal rate/%	0.002	+
			Greening coverage of built-up areas/%	0.021	+
		Society Development	Disposable income per urban resident/Yuan	0.034	+
			Total social consumer goods/Yuan	0.118	+

**Note**: "+" indicates a positive indicator; "-" indicates a negative indicator; "#" indicates a neutral indicator.

Within the evaluation index system of urban human settlement resilience, the positive indicator standardization method was applied to indicators with larger sample values that align closely with the requirements of urban human settlement resilience. Conversely, the negative indicator standardization method was employed for indicators that deviate from the desired direction [36]. Furthermore, with reference to existing literature studies [37], for the standardization of neutral indicators (e.g., annual average temperature, annual average relative humidity, and annual average light hours), we calculated the difference between each sample value and the mean value, and then adopted the negative indicator standardization. Finally, the comprehensive evaluation scores were calculated using the entropy value method weights.

1. “Pressure” indicators of urban human settlement resilience. "Pressure" refers to the unsustainable development factors of human activities that damage the urban environment. These factors contribute to the formation of the "state" and are also the result of the "response". In fact, urban human settlement constitutes a complex system involving urban resources, the natural environment, and human behavior, all of which collectively support the production and daily life of urban residents [38]. Given these considerations, the "Pressure" indicators selected to reflect urban human settlement resilience encompass the natural environment, living environment, and urban safety. Specifically, the natural environment indicators reflect the condition of the city’s natural environment, where a higher level of urban human settlement resilience corresponds to a better natural environment. The living environment indicators reflect the impact of human activities on the urban habitat, while the urban safety indicators relate to the safety of urban residents.
2. “State” indicators of urban human settlement resilience. "State" refers to the current state of the resource environment, which is the outcome of changes in the urban human settlement resilience under various "Pressures" and serves as the ultimate goal of "Response". In urban systems, the urban human settlement resilience against uncertainty relies on a diverse range of resources, including urban infrastructure, technology education, and health care [39]. Urban infrastructure indicators measure the level of urban development from a quantitative perspective, and the improvement of urban infrastructure contributes to enhancing the urban human settlement resilience. Technology education indicators serve as the foundation for enhancing a city’s innovation capacity and nurturing talent, reflecting the city’s level of innovation and its potential for development. Health care indicators reflect the level and coverage of medical and healthcare services in the city, which directly impact the well-being of its residents.
3. “Response” indicators of urban human settlement resilience. "Response" refers to the actions taken in response to specific "Pressure" and the current "State". Human regulatory behavior represents an active response to adapt to environmental changes, which can enhance the capacity of urban resources, alleviate pressure on the urban environment, promote social development, and have a positive impact on urban human settlement resilience [40]. Specifically, society input indicators reflect the extent of government financial support for education, science and technology, and pollution control. Environmental governance indicators contribute to creating a favorable ecological environment for the sustainable development of urban human settlement resilience. Society development indicators reflect the level of economic development in the city.

#### 3.4.2 Explanatory variables

Green development efficiency was selected as the core explanatory variable in this study, aiming to comprehensively reflect the level of green development in cities. According to the characteristics and development direction of green development in the YRD urban agglomeration, capital input, labor input and energy input were selected to measure the input level of regional green development with reference to the existing relevant studies [41,42]. In terms of input indicators, the indicator of the amount of social fixed asset investment was used to measure the capital input for the green development of the YRD urban agglomeration. Labor input was assessed based on the number of individuals employed in the region, while energy input was measured by the overall electricity consumption of society.

The measurement of green development output consists of two components: desired output and non-desired output. The desired output typically involves the measurement of total urban GDP, which serves as an indicator reflecting the level of regional development and plays a crucial role in assessing the regional economic development status. On the other hand, the non-desired output takes into account the negative impact on ecological environment in the development process with a wide range of pollutants, among which there are both similarities and differences [43]. In this study, the weighted average of industrial wastewater emissions, industrial sulfur dioxide emissions, and soot emissions from industrial exhaust gases are selected to reflect the non-desired outputs in the process of urban green development.

#### 3.4.3 Control variables

Analyzing the impact of green development efficiency on urban human settlement resilience requires considering the combined effects of multiple factors. With reference to relevant studies [44,45], population density, income level, urbanization rate, and industrial structure were selected as control variables for this study.

1. Population density (PD) refers to the number of individuals per square kilometer. According to the agglomeration effect theory, as population density increases, the urban economies of scale effect will progressively manifest, leading to the emergence of greater economic activities and innovation prospects [46]. This, in turn, generates an upswing in urban consumption demand and a bolstered labor supply for urban development, consequently fostering the refinement of green development efficiency and the elevation of regional urban human settlement resilience. It is worth noting that cities with high population densities often face challenges such as crowded living spaces, depleted infrastructure and energy constraints, all of which can negatively impact green development and urban human settlement resilience.
2. Income level (IL) is determined by the average wage of urban workers. It not only indicates the production and living conditions of urban residents, but also reflects the overall economic and social development of the city. Higher income levels can foster greater awareness and investment in environmental development, which in turn drives cities in a greener, more sustainable direction [47].
3. The urbanization rate (UR) is defined as the proportion of urban population to the total population, indicating the extent and pace of population aggregation in cities. According to Dang’s research, it was found that as the rate of urbanization increases, the space and population of cities continue to expand, putting more pressure on resources and ecosystems [48]. However, the higher urbanization rate also brings larger market scales and opportunities for innovation, providing a broader scope for enhancing urban human settlement resilience and promoting green development [49].
4. Industrial structure (IS) is measured by the share of tertiary sector output in the total GDP. According to the new economic geography, the industrial structure and economic attributes of cities influence their competitiveness and resilience [50]. The tertiary industry, characterized by its higher value addition, has the potential to generate numerous employment prospects and consumption avenues for the city, which means that the increase in the proportion of tertiary industry is conducive to realizing the balance between economic development and environmental protection, thus improving the efficiency of green development [51]. Meanwhile, the rapid growth of the tertiary industry facilitates the provision of high-quality services, cultural and recreational activities, attracting more skilled talents and fostering technological innovation, ultimately strengthening urban human settlement resilience.

## 4 Result analysis

### 4.1 Spatio-temporal evolution of urban human settlement resilience and green development efficiency

**Fig 4:**
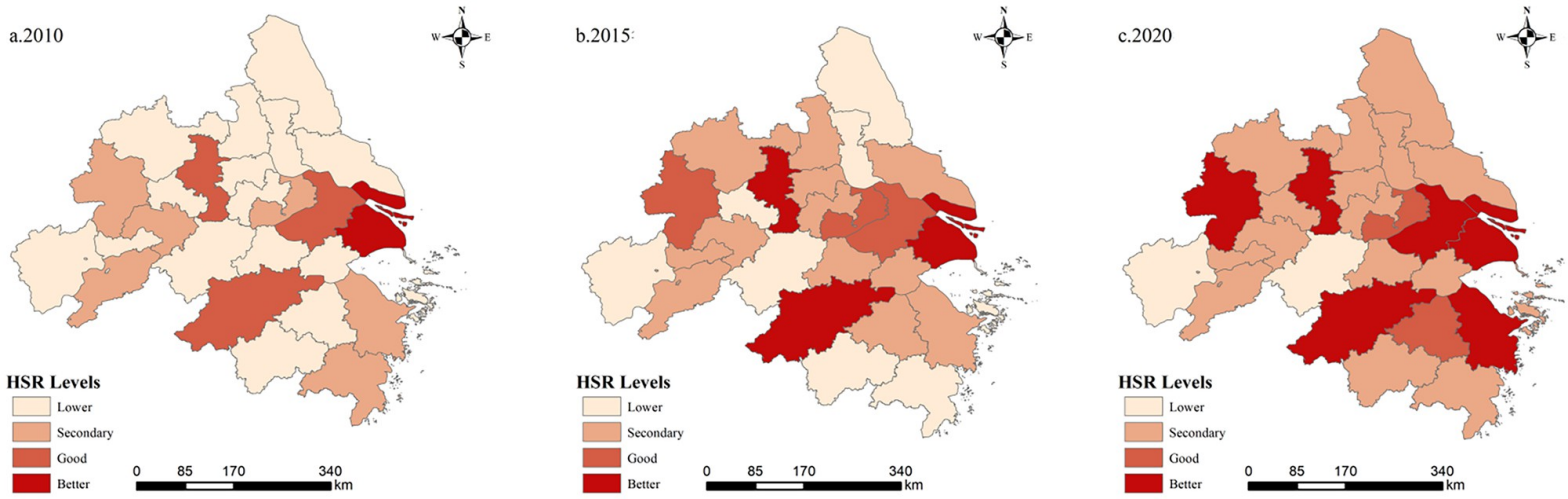
Spatial distribution of urban human settlement resilience.

**Fig 5:**
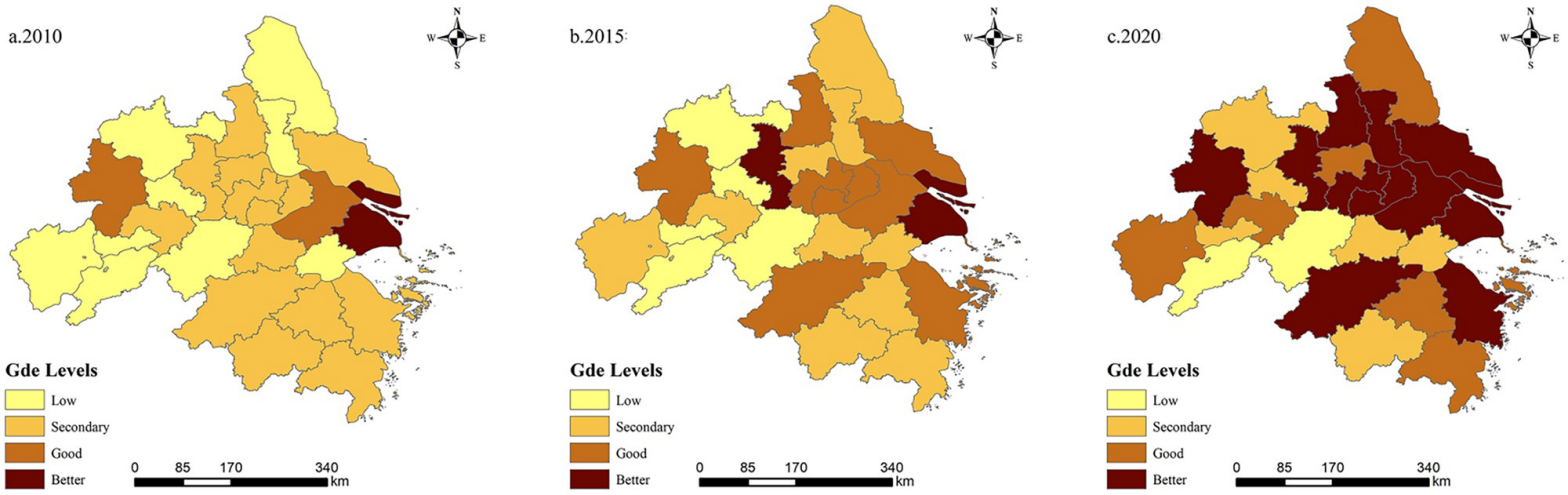
Spatial distribution of green development efficiency.

Based on the evaluation index system, the green development efficiency was measured using the super-efficient SBM method, and the combined values of urban human settlement resilience were assessed using the entropy value method for the period from 2010 to 2020 (Table 3). Over the past 11 years, the YRD urban agglomeration has experienced significant advancements in both green development efficiency and urban human settlement resilience. The values of green development efficiency and the levels of human settlement resilience for each city have shown a fluctuating upward trend over time, while the disparities between municipalities have been decreasing. To visualize the spatial and temporal evolution trends of green development efficiency and urban human settlement resilience in the YRD urban agglomeration, the levels of green development efficiency and urban human settlement resilience were classified into four categories using the natural breakpoint grading method. These categories were then mapped out using ArcGIS for three time sections: 2010, 2015, and 2020 (Figs 4 and 5).

**Table 3:** Level of green development efficiency and urban settlement resilience from 2010 to 2020.

Year	Gde 2010	Gde 2015	Gde 2020	Mean	Hsr 2010	Hsr 2015	Hsr 2020	Mean
Shanghai	0.40	0.53	1.12	0.67	0.43	0.49	0.64	0.50
Nanjing	0.26	0.41	1.02	0.47	0.37	0.44	0.57	0.45
Wuxi	0.27	0.34	0.51	0.36	0.30	0.33	0.36	0.33
Changzhou	0.22	0.29	0.39	0.30	0.23	0.28	0.30	0.27
Suzhou	0.28	0.33	0.54	0.37	0.30	0.38	0.52	0.39
Nantong	0.22	0.29	0.43	0.31	0.23	0.26	0.30	0.26
Yancheng	0.19	0.23	0.30	0.24	0.17	0.22	0.25	0.21
Yangzhou	0.24	0.31	0.44	0.33	0.20	0.25	0.27	0.24
Zhenjiang	0.23	0.27	0.34	0.28	0.22	0.26	0.27	0.25
Taizhou	0.18	0.25	0.40	0.27	0.17	0.19	0.24	0.20
Hangzhou	0.26	0.34	0.54	0.37	0.36	0.43	0.52	0.44
Ningbo	0.27	0.31	0.38	0.32	0.25	0.30	0.40	0.32
Jiaxing	0.18	0.21	0.27	0.22	0.21	0.24	0.30	0.24
Huzhou	0.21	0.24	0.27	0.24	0.22	0.25	0.29	0.25
Shaoxing	0.23	0.26	0.31	0.27	0.21	0.27	0.32	0.26
Jinhua	0.23	0.26	0.26	0.25	0.22	0.23	0.27	0.24
Zhoushan	0.25	0.32	0.34	0.36	0.17	0.20	0.23	0.20
Taizhou	0.26	0.26	0.32	0.27	0.23	0.23	0.29	0.25
Hefei	0.32	0.34	1.01	0.41	0.29	0.37	0.49	0.38
Wuhu	0.23	0.22	0.33	0.24	0.25	0.26	0.29	0.27
Ma’anshan	0.16	0.17	0.25	0.18	0.17	0.21	0.26	0.21
Tongling	0.18	0.16	0.21	0.20	0.16	0.24	0.23	0.22
Anqing	0.17	0.22	0.33	0.23	0.22	0.18	0.20	0.20
Chuzhou	0.20	0.20	0.26	0.23	0.22	0.26	0.26	0.24
Chizhou	0.14	0.15	0.20	0.16	0.24	0.26	0.26	0.24
Xuancheng	0.11	0.15	0.19	0.16	0.20	0.20	0.23	0.21

The spatial variation of urban human settlement resilience in the YRD urban agglomeration indicates an overall increasing trend from 2010 to 2020, but with significant spatial variation. It exhibits a spatial distribution pattern characterized by higher levels in the east and lower levels in the northern and southern edges. Specifically, the eastern region shows higher urban human settlement resilience compared to the central and western regions, while the core region, primarily Shanghai, demonstrates significantly better urban human settlement resilience than the surrounding areas.

With the passage of time, the spatial evolution of the YRD urban agglomeration has gradually transitioned from a "monocentric" distribution pattern with Shanghai as the core to a "polycentric" distribution pattern with Shanghai as the center and sub-centers including Hefei, Suzhou, Hangzhou, Nanjing, and Ningbo. In general, under the development tone of the integration of the Yangtze River Delta, neighboring areas such as Taizhou, Jinhua, and Yancheng have achieved stable growth and efficient development in terms of urban human settlement resilience over time. Moreover, cities such as Tongling, Chuzhou, Yancheng, and Yangzhou have also experienced significant improvements in urban human settlement resilience.

From 2010 to 2020, the green development efficiency of the YRD urban agglomeration has shown significant improvement. In terms of spatial distribution, the areas with high green development efficiency were mainly concentrated in the eastern coastal region, while Anhui still exhibited a development gap compared to Zhejiang, Jiangsu and Shanghai. However, with the introduction and implementation of the green development concept, particularly the deepening of the regional construction strategy, each region’s green development efficiency entered an accelerated stage. This led to the emergence of a "catch-up effect" in regional development, resulting in a narrowing of the differences in green development efficiency among cities. Although cities with high green development efficiency still exhibit a "patchy" spatial pattern, overall transformation of the YRD urban agglomeration from low-to-medium level dominance to a higher level has been achieved.

### 4.2 Analysis of the impact of green development efficiency on urban human settlement resilience

#### 4.2.1 Spatial correlation analysis

The global Moran’s I index was used to measure the spatial correlation of urban human settlement resilience and green development efficiency in the YRD urban agglomeration from 2010 to 2020. The results showed that the global Moran’s I indices were all greater than 0 and passed the significance test, indicating that urban human settlement resilience and green development efficiency were not randomly distributed in space and had significant positive spatial correlation characteristics (Table 4).

**Table 4:** The global Moran’s I index of green development efficiency and urban settlement resilience from 2010 to 2020.

urban human settlement resilience				Green development efficiency			
Year	Score	Year	Score	Year	Value	Year	Value
2010	0.184*	2016	0.224**	2010	0.333**	2016	0.386***
2011	0.256***	2017	0.231**	2011	0.419***	2017	0.328***
2012	0.257**	2018	0.214**	2012	0.405***	2018	0.237**
2013	0.254**	2019	0.198*	2013	0.434***	2019	0.271***
2014	0.253***	2020	0.232**	2014	0.442***	2020	0.134*
2015	0.25*			2015	0.412***		

**Notes**:

*, **, *** present the significance level of 10%, 5% and 1% respectively.

**Fig 6:**
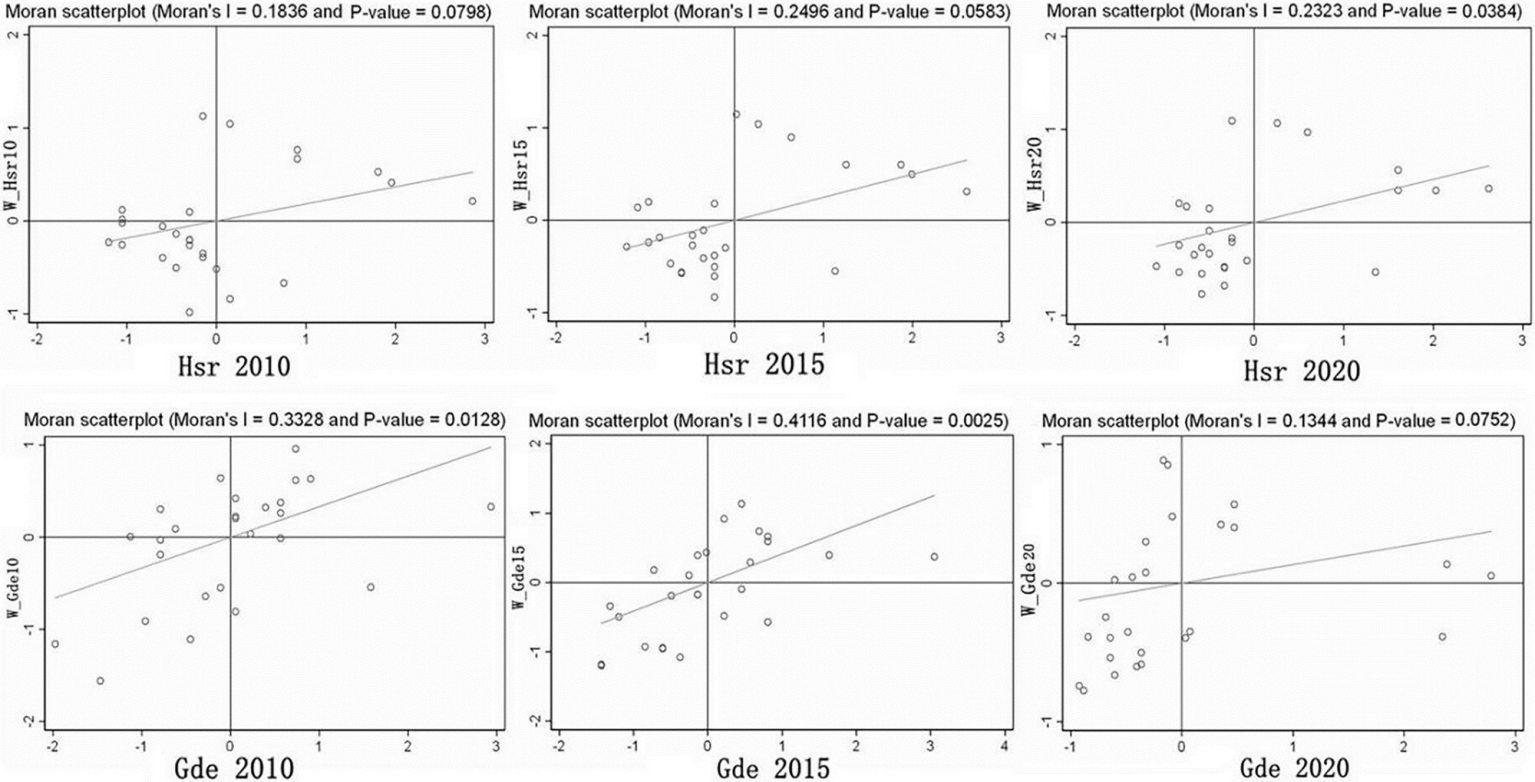
Moran Scattered plot of green development efficiency and urban settlement resilience in 2010, 2015 and 2020.

The global Moran’s I index confirms that there is spatial clustering in the distribution of urban human settlement resilience and green development efficiency within the YRD urban agglomeration. Furthermore, the local autocorrelation model is introduced again to specifically analyze the spatial distribution patterns of urban human settlement resilience and green development efficiency in the YRD urban agglomeration. Moran scatter plots for the years 2010, 2015, and 2020 are then generated (Fig 6).

Moran scatter plots are used to measure the degree of spatial variation in urban human settlement resilience and green development efficiency within urban agglomeration. As seen in Fig 5, a greater number of cities are positioned in quadrants 1 and 3 than in quadrants 2 and 4, indicating that the local spatial autocorrelation of urban human settlement resilience and green development efficiency in the YRD urban agglomeration is mainly manifested as H-H and L-L aggregation, and the spatial homogeneity is more prominent compared to heterogeneity.

#### 4.2.2 Selection of spatial econometric model

This study combines the two-step method proposed by Elhorst and the three-step method proposed by Elhors to test whether the SDM can be degraded to a spatial error model or a spatial lag model [52]. The model selection process involves the use of Lagrange multiplier (LM) tests in the form of LM-lag and LM-err, as well as robust R-LM lag and R-LM err tests (Table 5).

**Table 5:** Spatial econometric model test results.

Test of spatial econometric model	Statistics	P-value	Test of spatial econometric model	Statistics	P-value
LM-spatial lag	2.98	0.084	Wald-spatial lag	9.97	0.076
Robust LM-spatial lag	30.25	0.000	Wald-spatial error	14.01	0.015
LM-spatial error	3.701	0.054	LR-spatial lag	9.85	0.079
Robust LM-spatial error	30.97	0.000	LR-spatial error	13.71	0.017
Hausman	13.58	0.018			

The results indicate that the majority of the test statistics are significant at the 1% level, and all of them pass the significance test at the 10% level, indicating that the effect of green development efficiency on urban human settlement resilience has both spatial error and spatial lag forms. Additionally, Wald-spatial lag, Wald-spatial error, LR-spatial lag, and LR-spatial error tests were conducted and the findings reveal that the SDM cannot be simplified into SEM or SLM models. Consequently, the SDM was initially selected as the spatial econometric model for this study. To determine whether fixed or random effects should be employed, a Hausman test was conducted on the data. The results indicate that the null hypothesis was rejected at the 5% significance level.

Combined with the outcomes of the previous tests, it is evident that the time-fixed SDM is the most suitable model for measuring the impact of green development efficiency on urban human settlement resilience in the YRD urban agglomeration.

### 4.3 Analysis of the spatial durbin model regression results

#### 4.3.1 Analysis of model regression results

Before conducting the analysis of the spatial econometric model, we initially employed the Ordinary Least Squares (OLS) regression. It can be seen from the results in Table 6 that the spatial autoregressive coefficient of urban human settlement resilience is positive. This suggests a significant positive correlation between the human settlement resilience of cities, indicating that the local human settlement resilience of a city is influenced by neighboring cities with similar spatial characteristics.

**Table 6:** The regression results of the ordinary panel model and spatial Durbin model.

Explanatory variable	OLS	SDM	Decomposition of Spatial Effects		
			Direct effect	Indirect effects	Total effect
*Lngde*	0.063*** (4.00)	0.091*** (5.66)	0.086*** (5.14)	-0.105*** (-3.28)	-0.019 (-0.47)
*Ur*	0.268*** (5.34)	0.379*** (7.00)	0.371*** (6.98)	-0.110 (-1.02)	0.261** (2.08)
*Is*	0.196*** (3.22)	0.283*** (4.54)	0.287*** (4.80)	-0.041 (-0.41)	0.246** (2.27)
*Pd*	0.001*** (2.84)	0.001* (1.61)	0.001*** (1.84)	0.001* (1.67)	0.001** (1.94)
*Ic*	0.003** (1.90)	0.008*** (2.96)	0.008*** (2.99)	0.006 (1.33)	0.014** (2.12)
*WLngde*	——	-0.109*** (-3.88)			
*WUr*	——	-0.155* (-1.64)			
*WIs*	——	-0.075 (-0.77)			
*WPd*	——	0.001* (1.60)			
*WIc*	——	0.004 (0.99)			
*ρ*	——	0.165***			
Log-likelihood	——	483.0619			
N	286	286			
R^2^	0.7045	0.7994			

**Notes**:

*, **, *** present the significance level of 10%, 5% and 1% respectively.

Upon comparing the regression results of the ordinary panel model and the SDM, several observations can be made. Firstly, the OLS and SDM regression coefficients of green development efficiency, urbanization rate, industrial structure, population density, and income level are positive and also passed the significance test. This indicates that these five factors have a significant positive impact on enhancing urban human settlement resilience. Secondly, urban human settlement resilience exhibits spatial aggregation characteristics. The spatial autocorrelation coefficient under the time fixed effect passes the significance test at the 1% level, signifying that the level of local urban human settlement resilience is influenced by the levels of human settlement resilience in neighboring regions.

Lastly, it is important to consider that OLS regression models tend to overestimate the effects of explanatory variables on urban human settlement resilience because they neglect spatial spillover effects. Therefore, it is necessary to account for the cross-spatial effects of different regional explanatory variables on local urban human settlement resilience levels. The regression analysis results of each variable are as follows:

1. Explanatory variable. Analysis of the regression coefficient values reveals that the OLS regression and SDM regression coefficients of the core explanatory variable (LnGDE) are 0.063 and 0.091, respectively, and are significant at the 1% level. This indicates that green development efficiency can contribute to the improvement of the level of urban human settlement resilience. In contrast, the coefficient of the lagged term of LnGDE is negative and passes the significance test. This suggests that the green development efficiency of the region has a significant negative spatial effect on the neighboring cities’ human settlement resilience, possibly due to the "siphon effect" between regions. Specifically, on one hand, cities with high green development efficiency can attract more population, industries, and resources from neighboring areas, resulting in relatively slower development in the neighboring regions. On the other hand, the green development of a city requires significant capital and human resources, which are often sourced from neighboring areas, hindering the enhancement of urban human settlement resilience in those regions.
2. Control variables. Among the control variables, the majority of regression coefficients passed the significance test at the 1% level. Particularly, the regression coefficients of IC and PD, as well as their lagged terms, are significantly positive. These findings suggest that an increase in income level and population density generates positive spatial spillover effects, promoting the improvement of urban human settlement resilience in neighboring areas. Additionally, it is worth noting that the regression coefficients of UR and IS are positive, while their lagged term coefficients are negative. This indicates that these factors can have a positive impact on the regional urban human settlement resilience level but have a competitive relationship with neighboring areas.

#### 4.3.2 Endogeneity and robustness tests

1. Endogeneity Tests. There might be a mutual influence mechanism between urban human settlement resilience and green development efficiency, which raises concerns about endogeneity. Therefore, considering the possibility of endogeneity bias caused by reverse causality, this study explores the extent to which the endogeneity problem affects the results of the study by regressing the explanatory variables with a one-period lag, referring to the approach of Zhou and Yan [53,54].
2. Robustness Tests. In order to test the validity of the estimation results, two types of robustness tests were conducted: replacement of the spatial weight matrix and sub-sample regression [55]. Firstly, the economic weight matrix and the economic geographic weight matrix were used as replacements for the spatial weight matrix, and the Separate regressions were performed to compare whether there were significant changes in the results. As shown in Table 7, the model estimation results using the replaced spatial weight matrix were consistent with those of the baseline model, confirming the robustness of the SDM estimation results. Furthermore, regressions were conducted on a subset of the total sample, adjusting the sample time span to 2011 to 2019. The regression results and coefficients of the sub-sample were consistent with the baseline regression, as evident from the results in Table 6. This indicates that the SDM test results are robust.

**Table 7:** The regression results of the endogeneity and robustness tests.

explanatory variable	Endogeneity Tests	Robustness Tests		
	Lagged explanatory variables	Replace with economic distance matrix	Replace with economic geographic nesting matrix	Sub-Sample regression
*L_Lngdec*	0.092*** (5.00)	——	——	——
*Lngdec*	——	0.092*** (5.73)	0.094*** (5.90)	0.075*** (3.89)
*Ur*	0.448*** (7.77)	0.325*** (5.64)	0.361*** (6.93)	0.449*** (7.56)
*Is*	0.280*** (4.34)	0.273*** (4.48)	0.287*** (4.79)	0.253*** (3.64)
*Pd*	0.001 (1.32)	0.001* (1.91)	0.001** (1.93)	0.001 (1.20)
*Ic*	0.007*** (2.75)	0.009*** (3.60)	0.008*** (3.19)	0.006** (2.21)
*ρ*	0.1356**	0.1677**	0.2338**	0.1210
N	260	286	286	234
R^2^	0.8015	0.7811	0.7480	0.7852

### 4.4 Analysis of threshold model results

#### 4.4.1 Threshold effect test

Despite the confirmed significance of green development efficiency in enhancing urban human settlement resilience, its influence mechanism may also be affected by other variables, resulting in a non-linear relationship between them. Therefore, it is necessary to incorporate a threshold effect test model in this study. Building upon the previous econometric model and estimation method, this section selects population density and industrial structure as threshold variables to analyze the impact of green development efficiency on urban human settlement resilience at different threshold levels.

Before carrying out the threshold effect model analysis, the sampling was repeated 300 times using the Bootstrap method to test for threshold effects of the explanatory variables. The results are shown in Table 8. Specifically, the F-value for the single threshold test of population density is 66.53, indicating passed the significance test at the 1% level. Additionally, the F-value for the single threshold test of industrial structure is 20.89, demonstrating statistical significance at the 10% level.

**Table 8:** The results of the threshold effect test.

Variables	Type of models	F value	Bootstrap	Crit10	Crit5	Crit1
*IS*	Single threshold	20.89*	300	17.673	23.512	42.750
*PD*	Single threshold	66.53***	300	26.237	32.084	38.819

#### 4.4.2 Analysis of threshold regression results

Based on the threshold examination, the panel threshold model was selected to analyze the sample data, and the results are listed in Table 9.

**Table 9:** The results of the threshold regression.

**variables**	**Coefficient of estimation**	**T-value**	**P-value**
γ1: Is≤0.38	0.068	3.34	0.00
γ2: Is>0.38	0.080	4.14	0.00
*Ur*	-0.045	-0.74	0.46
*Pd*	0.001	1.25	0.22
*Ic*	0.010	7.09	0.00
*cons*	0.320	6.05	0.00
R^2^	0.7292		
F	41.33		
**variables**	**Coefficient of estimation**	**T-value**	**P-value**
γ1: Pd≤349.02	-0.068	-2.38	0.02
γ2: Pd>349.02	0.092	5.18	0.00
*Ur*	-0.165	-0.31	0.75
*Ic*	0.007	3.93	0.00
*Is*	0.038	0.46	0.64
*cons*	0.3	4.36	0.00
R^2^	0.7587		
F	39.19		

**Fig 7:**
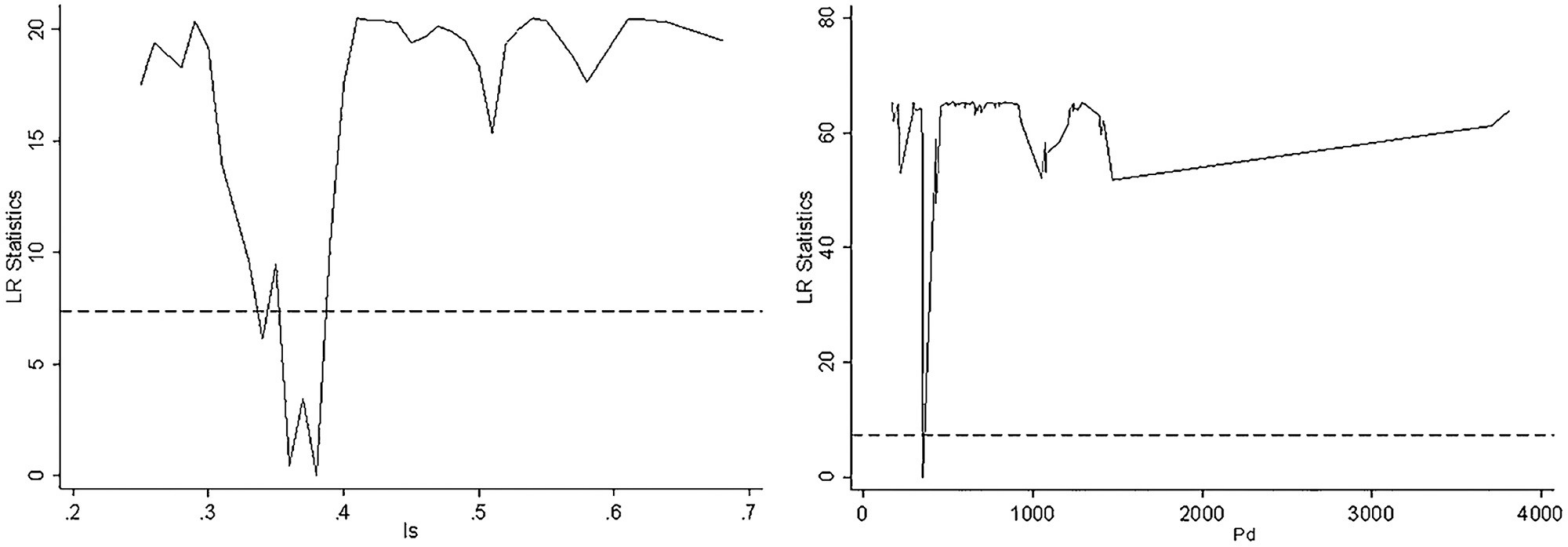
Threshold value and confidence interval of panel threshold model.

(1) population density (PD). The impact of green development efficiency on urban human settlement resilience at different population density levels shows a significant "V" type non-linear characteristic. When PD is less than or equal to 349.02 (PD ≤ 349.02), the regression coefficient is negative, indicating that the increase in green development efficiency at low population density levels does not have a significant promotional effect on the development of urban human settlement resilience. After PD exceeds 349.02 (PD>349.02), the regression coefficient changes from negative to positive, and the optimization of green development efficiency positively promotes the improvement of urban human settlement resilience. (Fig 7)

This could be attributed to the challenge of creating a "scale effect" in the provision of public services at low population densities, as well as the greater pressure faced by cities in maintaining public infrastructure, resulting in relatively weak construction and management of the human and natural environment, which hampers the increase in the level of urban human settlement resilience. However, as population density increases, the concentration of people not only drives the growth of consumption demand but also provides the necessary labor supply for urban development. This, in turn, directs more attention toward improving urban functions. In such cases, optimizing green development efficiency will have a noticeable positive effect on increasing the level of human settlement resilience.

(2) Industrial structure (IS). The effect of green development efficiency on urban human settlement resilience increases in a stepwise manner at different IS. Specifically, when IS is less than or equal to 0.38 (IS≤0.38), the promotion of green development efficiency on urban human settlement resilience is slow, while when IS exceeds 0.38 (IS>0.38), the optimization of green development efficiency becomes significant in promoting the improvement of urban human settlement resilience.

This finding indicates that, firstly, the characteristics of regional industrial structure largely determine the pattern of resource consumption and pollution emission in the region. Secondly, the transition from a primary industry to secondary and tertiary industries can better facilitate a win-win situation for urban economic and social development, as well as ecological environmental protection. This transition also improves the environmental comfort and social convenience of urban residents’ lives. Therefore, with the rise of the proportion of tertiary industry in GDP, the promotion of green development efficiency to the level of urban human settlement resilience is more obvious.

## 5 Discussion

Since the reform and opening up, China has embarked on a large-scale urbanization process and achieved leapfrog development, which requires a constant renewal of urban connotation. From the evolution of the concept of urban human settlement resiliencet, it can be seen that residents generally recognize that a modern city should include a natural ecological environment suitable for living and a convenient humanistic environment [56]. In this context, the introduction of the concept of green development has further provided ideological guidance for improving urban human settlement resilience, which achieves sustainable urban development by promoting the realization of the unity of economic, social and environmental benefits. So, what can we learn from this study?

1. It is necessary to promote the development of a green and livable city with a "multi-core" drive. As key drivers of growth in the YRD urban agglomeration, megacities play a central role in facilitating coordinated regional development and ensuring a balanced allocation of regional resources [57]. With the deepening development of urban agglomeration, core cities become more competitive in terms of economic development and innovation capacity, while surrounding cities face greater development pressures and challenges due to relatively fewer resources and a more homogenous industrial structure [58]. This situation gives rise to the problem of unbalanced regional development and suggests that the driving model of a single core city is not sufficiently flexible and sustainable. As a result, the integrated development strategy of the YRD has been upgraded to a major national economic development strategy, and the YRD urban agglomeration has begun to transform into a "multi-core driven" model with the joint efforts of a number of core cities.
2. Construction of livable cities needs to rationally avoid the siphoning effect between cities. The results of SDM show that the green development efficiency of the region within the YRD urban agglomeration presents a significant negative spatial spillover effect on urban human settlement resilience in the neighboring regions, suggesting a possible siphoning effect between regions [59]. From the perspective of the urban development process, core city development will be accompanied by the centralized relocation of resources and industries, which may result in the outflow of resources and economic marginalization of the surrounding cities, and thus not be conducive to enhancing the level of urban human settlement resilience within them [60]. Thus, it reveals that neighboring cities can formulate differentiated development strategies and lay out well-developed transportation networks during urban development to meet the challenges.
3. Selecting appropriate development models based on a comprehensive understanding of local conditions. The YRD urban agglomeration, as one of the most dynamic and development potential regions in China, has complex economic linkages and resource complementarities among its cities [61]. According to the results of the threshold model, with the increase of population density and the optimization of industrial structure, the impact of green development efficiency on the urban human settlement resilience increases in the form of a "V" shape or a staircase within the YRD urban agglomeration. This reveals that we need to have an in-depth understanding of the industrial structure and economic characteristics of different cities within the YRD urban agglomeration, which have their own strengths and characteristics in terms of industries, and that the government should look for the advantageous industries and develop and expand them.

## 6 Conclusions and policy implications

At present, the construction of habitat environment is facing new challenges, and the concept of sustainable development is deeply rooted [62]. Therefore, it is necessary to introduce the theory of resilience into urban habitat environment and enrich the theory of habitat environment. Previous studies had rarely put green development and urban human settlements together, and literature on the mechanisms of green development’s impact on urban human settlement resilience was even scarcer. Consequently, the mechanism and intensity of the impact of green development on urban human settlement resilience in the YRD urban agglomeration, as well as the spatial spillover and threshold effects of such impacts, are the starting point and core theme of this study.

### 6.1 Conclusions

Based on the measurement of green development efficiency and urban human settlement resilience of 26 cities in the YRD urban agglomeration from 2010 to 2020, this study reveals their spatio-temporal patterns and development characteristics. Moreover, we further investigates the spatial spillover effect of green development efficiency on urban human settlement resilience and the non-linear influence relationship between them under different population density levels and industrial structures by establishing a SDM and a threshold panel model, the results of the study show that:

1. During the study period, the levels of green development efficiency and urban human settlement resilience in cities within the YRD urban agglomeration experienced rapid growth. The spatial evolution of urban human settlement resilience level is characterized by a "monocentric" distribution pattern gradually expanding into a "polycentric" distribution pattern, as well as a "speckle-like" distribution of high green efficiency cities in space.
2. From the spatial correlation results, it can be seen that there is a strong positive spatial correlation between green development efficiency and urban human settlement resilience level, which indicates that spatial agglomeration tends to be formed between cities with higher and lower levels. Meanwhile, the Moran scatter plot mainly shows H-H and L-L aggregation, and spatial homogeneity is more prominent compared to heterogeneity, indicating that future analyses should take spatial effects into account.
3. According to the validation of LM, LR, Wald and Hausman models, SDM was the most suitable model to be used as a measuring model for the impact of green development efficiency on urban human settlement resilience in YRD urban agglomerations. Specifically, the results of SDM show that the spatial autoregressive coefficients are significantly positive, proving that urban human settlement resilience of each region was affected by other similar spatial characteristics of neighboring regions human settlement resilience in the YRD urban agglomeration. Moreover, the green development efficiency of each city in the YRD urban agglomeration has a significant contribution to urban human settlement resilience in the region, but has a negative spatial effect on the level of urban human settlement resilience in the neighboring region.
4. Using the threshold model, we found that under different population density levels, the effect of green development efficiency on urban human settlement resilience shows a significant "V" nonlinear characteristic. In addition, the influence of green development efficiency on urban human settlement resilience increases in a stepwise manner under different industrial structure distribution, demonstrating there was a threshold effect of green development efficiency on urban human settlement resilience under different threshold variables.

### 6.2 policy implications

1. In the process of urban construction, the YRD urban agglomeration needs to achieve integrated development and solve the problem of unbalanced development, and urban development should establish a mechanism of close coordination between cities to promote the complementarity and transformation of resource advantages. Furthermore, the communication and cooperation between cities should be emphasized, and the interconnection of advantageous industries across cities should be promoted, so as to promote the synergistic development of cities, which can ultimately maximize the overall benefits of urban agglomeration.
2. In the process of urban development, it is essential to consider transforming the economic development model and vigorously promoting the concept of green development. The YRD urban agglomeration needs to emphasize green development transformation with enhanced provision of an ecologically green and beautiful environment [63]. Specifically, it should promote the construction of urban green spaces, improve the quality of life for residents and the resilience of the ecological environment, thus striving to create a new type of modernized cities that complement the upgrading of urban development and the harmony and livability of the environment.
3. In the process of cooperation between cities, it is important to explore different models of development. The government should recognize the industrial structure and economic characteristics of different cities within the YRD urban agglomeration, and identify advantageous industries and innovative resources to provide strong support for green development. At the same time, attention should be paid to the living needs of residents, and new opportunities should be created through the development of green industries, resulting in enhanced urban human settlement resilience.

## 7 Research limitations

Although this paper preliminarily discusses the development level and influencing factors of urban human settlement resilience by analyzing the spatial spillover and threshold effect of green development efficiency on urban human settlement resilience, there is still a large scope for improvement.

Firstly, based on the great strategic position of the YRD urban agglomeration in the overall situation of national modernization and the all-round opening up pattern [64], we choose it as the research area. However, considering that the YRD urban agglomeration is a unique and vibrant area in China, the applicability of the research findings and illumination in other late-developing areas still needs to be verified by further research.

Secondly, as a multi-dimensional concept, the construction of the evaluation index system of urban human settlement resilience is restricted by data availability, measurability and other factors, leading to the connotation of urban human settlement resilience may not be able to be comprehensively interpreted and measured. Therefore, indicators such as ecological service value and land-use pattern can be measured in combination with natural data in the future to more comprehensively assess and measure the resilience of human settlement.

Finally, this paper also finds that the impact of green development efficiency on urban human settlement resilience has heterogeneity and threshold effects due to different population density and industrial structure. How to determine the optimal urban population size and industrial structure from the point of view of livable resilience is thus a topic worthy of further debate.

## Supporting information

S1 File (XLSX)
